# Supplementary material for: Plant attribute extraction: An enhancing three-stage deep learning model for relational triple extraction
Source: PLoS One. 2025 Jul 8;20(7):e0327186. doi: 10.1371/journal.pone.0327186 (PMC12237039; doi:10.1371/journal.pone.0327186)
Supplement: Attachment — Submitted filename: response_to_reviewers.docx [file pone.0327186.s001.docx]

Dear Editors and Reviewers,

We would like to thank you very much for your valuable comments and good suggestions that greatly help to improve our manuscript. We have carefully considered your valuable comments and good suggestions. In the following, we are going to explain how your comments have been taken into full account in the revision.

#reviewer 1, Comment 1: The methodological description, while detailed, seems to gloss over specific challenges associated with the tagging-based joint extraction method. There is a noticeable lack of discussion about the potential pitfalls or limitations of parameter sharing, which could be detrimental to the model’s applicability.

#Response: The specific challenges related to tagging-based joint extraction methods are supplemented in the related work section. The literature related to improvements in parameter sharing has been supplemented. The narrative logic of the related work section is as follows. Transitioning from describing the pipeline models to describing the joint extraction models. In the description of the joint extraction models, the tagging-based decoding strategy is introduced first, followed by the parameter sharing method, and finally, the extraction work related to the plant texts is introduced. Four pieces of literature are selected for discussion in the context of tagging-based methods, and the issues encountered by the methods in this literature are explained. The binary sequence labeling is analyzed with emphasis. We have listed three pieces of literature related to parameter sharing and identified the issues they address. Finally, the improvements made to our model are described.

#reviewer 1, Comment 2: The concept of breaking down the task into three stages for complexity reduction is not entirely novel, as similar methodologies have been used in other fields of data extraction and processing. The manuscript lacks a detailed comparison with these existing methods, making it difficult to gauge the novelty or superiority of the proposed “Bwdgv” method. The statistical evidence provided to support the claims of improved performance over other models like PRGC is insufficient. More rigorous testing and validation are needed to substantiate these claims credibly.

#Response: Joint extraction of entities and relations is mainly divided into span-based, table-filling, and tagging-based methods. We explain the categories of extraction in the paper and point out that our model belongs to the tagging-based method. Our work focuses on the interaction between tasks, including the adjustment to the word embedding and the optimization in relation prediction. Most of the models to compared have the characteristic of multi-tasking. Many models based on tagging-based are multi-task models. Some extract the head entity and tail entity separately, some extract the head entity and tail entity simultaneously, and others extract the tail entity and the relation simultaneously. To make it easier for readers to understand, we describe the task by directly dividing it into three parts. When looking for comparison models, we primarily choose the latest tagging-based models. By comparing with these latest models, we evaluate the superiority of our model.

The experiment in this article is mainly explained from the following aspects:

(1)When analyzing why our model outperforms other models, models are evaluated based on metrics such as precision (prec), recall (rec), and F1 score.

(2) We replicate the current state-of-the-art models based on the plant dataset and supplement the CMeIE dataset to validate the model's performance.

(3) We conduct ablation experiments on the datasets, removing the component of adjustments to the word embedding and the component of optimization in relation prediction respectively to validate the model's performance.

(4) We compare the changes in metrics during training for different models.

(5) The overlapping triples are categorized into Normal, SEO, SOO, and EPO. We analyze the proportion of these overlapping triples and compared the accuracy (prec), recall (rec), and F1 score (F1) of different models under the overlapping triples.

(6) We analyze the impact of the number of different triples in the input sentence on the results. The triples are mainly categorized into cases of N=1, N=2, N=3, N=4, and N≥5. We compare the accuracy (prec), recall (rec), and F1 score (F1) of different models under overlapping triples.

#reviewer 1, Comment 3: The manuscript suffers from numerous grammatical errors and awkward phrasing. The use of technical jargon is excessive without adequate explanation, which might alienate readers who are not deeply familiar with the field.

#Response: We have organized the entire text and made grammatical revisions from beginning to end. The paragraphs have been carefully modified. We have explained the terms as much as possible and reduced the use of excessive technical jargon.

#reviewer 1, Comment 4:

(1) The figures and tables, while informative, are sporadically discussed in the text. Some figures seem to be referenced out of order, which can confuse the narrative flow of the document.
(2) The technical section lacks a critical analysis of why the Bwdgv model performs better under certain conditions. The discussion about the model's performance in real-world scenarios or its scalability is notably absent, which is a significant oversight for a research article claiming substantial improvements in extraction methodologies.
(3) The explanation of the word embedding adjustments and their specific impacts on the model’s performance is too vague. Detailed examples or case studies demonstrating these impacts would lend more credibility to the authors' assertions.

#Response:

(1) We have rewritten the analysis of the experimental section (4.2). We organize the figures and tables, and provide detailed descriptions for each.
(2) We have rewritten section 4.2.2. We have listed the performance metrics of the model under different overlapping patterns and provided the proportions of patterns for analysis. We conduct a critical analysis of the model's performance regarding the four overlapping patterns: Normal, SEO, EPO, and SOO. Additionally, we provide an analysis of the model's strong performance in the SEO pattern. We then validate the model's performance with different numbers of triples and analyze why the model performs better as the number N of triples increases. Finally, we select sentences to showcase the actual extraction results and propose suggestions for improving the model based on its performance.
(3) We have rewritten section 4.2. In subsection 4.2.1, we analyze the impact of the word embedding adjustments through ablation experiments. In subsection 4.2.2, we also provide a brief discussion on the scenarios of overlapping patterns and the number of triples.

#reviewer 1, Comment 5: The manuscript does not address data sharing adequately. The sources of the datasets are mentioned, but there are no links or references where these datasets can be accessed, nor is there sufficient detail on the preprocessing steps taken.

#Response: At the end of the article, in the Data Availability Statement, we provide links to the datasets and add explanations about the dataset in Section 4.1.

#reviewer 2, Comment: sorry the manuscript is not suitable for this journal. The manuscript under title: Plant Attribute Extraction Based on an Enhancing Three-Stage Model” is just like report. I can’t consider it for publication. There is no innovation to contribute to the scientific community.

#Response: We have standardized and revised the format and grammar of the entire text, writing it according to the requirements of a research paper. The paper is organized into five sections. Section 1 provides an introduction. Section 2 presents a review of the current literature on relation extraction, outlining the strengths and weaknesses of existing approaches. In Section 3, we provide a detailed description of the workflow and various subtasks of the model. Section 4 focuses on the analysis of experimental data to validate the effectiveness of our proposed model. Finally, in Section 5, we summarize the work and propose directions for future improvement.

Sincerely,

Zhihao Zong
