# Supplementary material for: Plant attribute extraction: An enhancing three-stage deep learning model for relational triple extraction
Source: PLoS One. 2025 Jul 8;20(7):e0327186. doi: 10.1371/journal.pone.0327186 (PMC12237039; doi:10.1371/journal.pone.0327186)
Supplement: Attachment — Submitted filename: Response to Reviewers.docx [file pone.0327186.s003.docx]

Reviewer #1: I would say this manuscript is publishable after major revisions. The reasons are as follows:

1). Formula labeling inconsistency. The third section contains inconsistent labeling of the same formula within the text, which may lead to reader confusion and negatively impact the article's readability.

Answer:

(We revised formula4 and the text：where g is the derivative of L(x+∆x, y;θ) at the word embeddings.）

2). Inadequate explanation of attention mechanism. While Section 3.4 mentions the attention mechanism, it lacks a detailed explanation of its specific implementation and how the computed weights influence the model's prediction process.

Answer:

(We have added an explanation of the attention mechanism, and briefly explained how weights are assigned and the benefits of weight assignment.

The corrected part starts from line 488:

where the text vector and relation vector are directly concatenated to form a new composite vector. This operation allows the model to consider both the overall text information and specific relational information, thereby enhancing the accuracy of subject and object identification. Next, we use the attention mechanism to allocate weights to information, dynamically adjusting the model's focus on different pieces of information. The self-attention mechanism is adopted as the input consisted only of concatenated composite vector. For this composite vector, we use different linear transformations to generate the Query vector, Key vector, and Value vector. These transformations are usually fully connected layers, which map the input to different dimensional spaces. By computing the dot product between the Query vector and Key vector, a score matrix is obtained, reflecting the relevance of each element to all others. Then, to characterize the importance of the current information, the softmax function is used to convert the score matrix into a weight matrix. The weight matrix is finally used to perform a weighted calculation with the Value vector, resulting in the output vector for the position. After these steps, the text vector and the relation vector can be better integrated to highlight important information.）

3). Limited dataset scale. The current dataset size is relatively small, with an insufficient number of samples, potentially compromising the model's generalization capability and the reliability of the experimental findings. It is strongly recommended that the authors expand the dataset and conduct additional experiments to validate the model's performance and robustness.

Answer:

(We acknowledge that the current dataset size is relatively limited, which is indeed a major limitation of this study and may affect the direct generalizability of the results. As you pointed out, accurately evaluating model performance typically requires a larger dataset. Regarding the dataset, we have conducted preliminary calculations. Since the dataset requires manual precise annotation, especially the marking method of entity relations needs meticulous judgment and verification. Based on our experience, approximately 30 pieces of data can be processed per day on average. This annotation process is not only time-consuming but also requires high professional knowledge and meticulousness from annotators to ensure the accuracy of entities and their relations. Considering the time required to revise the paper and my personal work needs, we apologize that we can only supplement a few hundred data points within a short period (e.g., one month). At the current stage, there are indeed significant time challenges in supplementing a large-scale dataset.

We fully understand your concerns about the potential impact of dataset size on the reliability of the evaluation. To present our work as rigorously as possible and enhance the credibility of the results, we have added the following content in the revised manuscript.

1. Added comparative experiments: We introduce two relevant advanced models (GRTE and UniRel) as new baselines for comparison to more comprehensively evaluate the effectiveness of our proposed method. The compared models are shown in table 3.

2. Added ablation study: For the key perturbation term Δx in the model, we conduct a more detailed quantitative analysis by setting different values of Δx to verify the changes in model performance, demonstrating the rationality of our method design. The modified part is shown in the part of 4.2.2. Ablation Study.

3. Added a description of the CMeIE dataset: The dataset contains 14,339 training data samples. Which is one of the most diverse and publicly available datasets, ensuring the reliability of the model’s results. The description is show in the end of the part 4.2.1. Overall Results.

We recognize that small-scale datasets indeed have inherent limitations. In specific research fields or exploratory work, researchers sometimes conduct preliminary validations under limited data scales to demonstrate the feasibility of new methods or ideas. For example, the commonly used dataset in SOTA, WebNLG, has a training data size of 5,019. Although the current scale of our dataset limits the direct generalization ability of the results, it provides a basis for us to verify the effectiveness of the model. We hope that the reviewers can understand the efforts we have made and the difficulties in supplementing large-scale datasets at the current time point. We will improve the dataset in subsequent research.)

4). Suboptimal visual presentation. Several figures and tables are either excessively dense or poorly organized, diminishing their readability and overall visual effectiveness.

Answer:

(

1. We merge table 2 and table 3 into one table for description, and rename it to table 3. This table only describes the comparison results of the experiment. The original table 3 is replaced by table 4, which only describes the ablation experiments. Separating the comparison experiments and ablation experiments, which enhances the readability of article.

2. We add description between Table 3 and Figure 8. Considering the conciseness of the article and that these formulas do not affect the description, we removed original formula 1, formula 2, and formula 5.

3. We adjust the position of Figure 12, moving it to Section 4.1 Experimental Data and Setting to enhance the readability of the summary in 4.2.3 regarding different triplet patterns. In the section 4.1, we provide an explanation for SEO.)

Reviewer #2:

1.It is recommended that the authors reorganize the content by dedicating a separate section to the ablation study. This would allow for a more systematic and in-depth analysis of each module's contribution to the overall model performance, thereby enhancing the clarity and scientific rigor of the manuscript.

Answer:

(We add a description of the comparison between different models and make the ablation experiments as a separate section. The corrected parts are in the section 4.2.1. Overall Results the section and the section 4.2.2. Ablation Study)

2.The methodology section in Chapter 3 lacks comprehensive explanations for some formulas. Key variables and derivation processes are not adequately clarified. For instance, while Formula 5 describes the distance between vectors, it fails to elaborate on how this distance is utilized in adversarial training. Similarly, Formula 7 outlines the calculation of disturbances but omits the specific meaning and computational steps of the involved variables. Providing these details is crucial for readers to fully understand the technical foundations of the proposed approach.

Answer:

(

$$d=\sqrt{\left( x1-x2 \right)^{2}+\left( y1-y2 \right)^{2}+\ldots+\left( n1-n2 \right)^{2}}$$

1.

The formula, is used to illustrate that after text is converted into vectors, the distance between two vectors is constant and it is inappropriate to add a perturbation Δx to this vector. Other methods should be considered. To prevent misunderstanding that this is the method adopted in this paper, the standalone formula should be removed. Thus, We explain what a one-hot vector is and emphasize that adding perturbations after converting text into vectors is ineffective.

The corrected part starts from line 354:

The vectors converted from text are essentially one-hot vectors. A one-hot vector has a value of 1 at only one position and 0 everywhere else. The distance between the any two vectors is always √2. Therefore, adding perturbations to text vectors also has no effect.

$$\min_{\theta} E\left( x,y \right)\sim D\left[ \max_{\Delta x\in\Omega} L\left( x+\Delta x,y;\theta\right) \right] \left( 3 \right)$$

$$g=\nabla xL\left( x+\Delta x,y;\theta\right)$$

$$\Delta x=\varepsilon\frac{g}{{||g||}^{2}} (4)$$

2.

Formula (7) is defined below as Formula (4). The L function used in Formula (4) originates from Formula (3). We correct the formula and state that g is the gradient of the L function at the embedding. The L function can be understood as the entire operation process of the model. The parameters of the L function are explained in Formula (5). Finally, the chosen perturbation is the normalized value of g. The comprehensive improvement explains how to obtain the perturbation Δx and how to calculate the overall process.

The corrected part starts from line 381:

The core of adversarial training is to find an appropriate ∆x. Word embedding layer, as the direct interface between the model and input text, serve to map words into dense vectors. Adding perturbations here can directly affect the model’s understanding of vocabulary. In addition, compared to adding perturbations in deeper layers, perturbations in the word embedding layer are easier to control and do not have impact on the other structures of the model. Thus, we add perturbations in the word embedding layer. As shown in Fig. 3.

**Figure 3.** Word Embedding layer with perturbations.

The perturbation ∆x is directly added to the word embedding matrix. The input is first converted into a one-hot vector, and then transformed into a text vector through the word embedding layer. At this point, different samples will share the same perturbations, making the implementation easier. Finding the location where the perturbations need to be added, and then the perturbations can be calculated. The goal of perturbations $\Delta x$ is to maximize $L(x+\Delta x,y;\theta)$, and the direction of gradient ascent for $L$ is exactly the direction in which $L$ increases. We view the gradient of $L$ to be proportional to $\Delta x$. The calculation of the perturbations is shown in Equation (4)

$$g=\nabla xL\left( x+\Delta x,y;\theta\right)$$

$$\Delta x=\varepsilon\frac{g}{{||g||}^{2}} (4)$$

where g is the derivative of $L(x+\Delta x,y;\theta)$ at the word embeddings, $\varepsilon$ is the proportional coefficient, normalization is used to prevent perturbations from becoming too large. When constructing perturbations, the first step is to compute the loss of forward propagation and record the gradients of backward propagation. At this point, gradients obtained from word embeddings are regularized to obtain perturbations. And then, the perturbations are added to the word embedding to construct a new input $x+\Delta x$. Finally, the loss of forward propagation and the gradient of backward propagation are calculated once again. It should be noted that in the first training, the gradient does not need to be updated. In the second training, the gradient to be updated is the sum of the gradients calculated in the first and second computations. At this time, the model learns the features of both normal samples and adversarial samples simultaneously, better adapting to data changes and reducing fluctuation in the training. When updating the gradient, the input of word embeddings should revert to the original $x$. As the update without considering disturbances, the model reduces its reliance on adversarial samples, ensuring the stability of parameters. Based on this, the model learns how to resist noise at the input level and ensures the stability of the training process.

)

3.Although the proposed model demonstrates certain advantages in the comparative experiments, the number of baseline models included in the comparison is relatively limited. Expanding the comparison to include more state-of-the-art models would strengthen the validity and persuasiveness of the experimental results.

Answer:

(We increased the number of models and conducted a comparative analysis. The corrected part is in the section 4.2.1 Overall Results.

The corrected part starts from line 643:

GRTE improves global features based on TPLinker, but has low decoding and inference efficiency. Compared with these two, Bwdgv only constructs a matrix in entity pair matching, and optimizes entity recognition, enhancing its generalization ability.

UniRel focuses on the integration of relational semantics, achieving mutual inference between entities and relations. In comparison, Bwdgv not only focuses on relations but also considers the balance between tasks.）

4.Section 3.3 mentions the dimensions of the global matrix and the prediction method but does not provide a clear explanation of how the global matrix is constructed. A detailed description of this process is essential for reproducibility and for readers to fully grasp the methodology.

Answer:

(We were not clear enough in our description of the global matrix, and did not specifically indicate that the result calculated from formula (6) is the representation form of the global matrix. We have revised the narrative style and rewritten how to understand the global matrix and how to construct the global matrix.

The corrected part starts from line 459:

The extraction of plant entities and plant attributes along with the classification of plant attributes can be performed simultaneously. As shown in Fig. 1, we use a global matrix to predict plant entities(subjects) and plant attributes(objects) in the phase of entity pair extraction. The dimension of the global matrix is determined by the number of tokens in the sentence. If there are n tokens, the shape of the matrix is R^(n×n). The columns of the global matrix represent the starting positions of the subjects, and the rows represent the starting positions of the objects. The elements in the global matrix indicate whether the subjects and objects begin from these positions. To obtain the global matrix, the tokens of the subjects and objects are first concatenated vertically. Then, a fully connected layer is used to compute the concatenated information. The value of each element in the global matrix represents the confidence between a subject and an object. The higher the confidence, the more likely the begin position will be predicted. The extraction of plant entities and plant attributes remains a classification task, thus we use the sigmoid function to predict the begin positions of subjects and objects. The resulting prediction is the global matrix, which can be expressed as Equation (6).)

Reviewer #3:

1) Title can be improved by sharing approach names like adding terms like deep learning etc for better understanding of the domain

Answer:

(We have revised the title to make it more specific. The corrected title is: Plant Attribute Extraction: An Enhancing Three-Stage Deep Learning Model for Relational Triple Extraction.)

2) “Framework of Model” heading does not convey enough information. Is it the proposed model?

Answer:

(Yes, this heading refers to the proposed model. We change the heading “Framework of Model” to “The Framework of the Bwdgv Model”.)

3) Equation 5 presents the distance of the vector. However the equation is very generic and should be presented by changing symbols as per the domain in view on which the paper is written not generic symbols.

Answer:

($d=\sqrt{{(x1-x2)}^{2}+{(y1-y2)}^{2}+\ldots+{(n1-n2)}^{2}}$。

This formula is used to illustrate the calculation of distances between one-hot vectors. However, this formula is general and relatively simple. Considering that this paper only explains that it is not suitable to add perturbations to vectors, and it is not the method of adding perturbations used in this paper, we remove this formula and directly describe it in the text.

The corrected part starts from line 351:

The text representations are used as input for subsequent subtasks. Considering the accuracy of the information and the stability of the training process, perturbations are introduced into the model. Perturbations can be implemented through data augmentation, but text data does not have operations like image flipping or cropping. The vectors converted from text are essentially one-hot vectors. A one-hot vector has a value of 1 at only one position and 0 everywhere else. The distance between the any two vectors is always √2. Therefore, adding perturbations to text vectors also has no effect.)

4) The related work section is poorly written and managed. Only 4 references are discussed and then whole framework of ETL is presented with detailed mathematical background. It is recommended that the related work should present the review of existing studies. Latest papers published in top journals should be discussed. Few are given for your guidance, these and more to be added. Also, try to add another heading and share ETL and equations and then equations of BiLSTM should be presented in that new heading.

Guo, X., Zhu, Y., Li, S., Wu, S., & Liu, S. (2025). Research and Implementation of Agronomic Entity and Attribute Extraction Based on Target Localization. Agronomy, 15(2).

Dalvi, P., Kalbande, D. R., Rathod, S. S., Dalvi, H., & Agarwal, A. (2024). Multi-Attribute Deep CNN-Based Approach for Detecting Medicinal Plants and their Use for Skin Diseases. IEEE Transactions on Artificial Intelligence.

Wang, M., Zhang, S., Li, R., & Zhao, Q. (2024). Unraveling the specialized metabolic pathways in medicinal plant genomes: A review. Front. Plant Sci, 15, 1459533. doi: https://doi.org/10.3389/fpls.2024.1459533

Chen, C., Wang, R., Chen, M., Zhao, J., Li, H., Ignatieva, M.,... Zhou, W. (2025). The post-effects of landscape practices on spontaneous plants in urban parks. Urban Forestry & Urban Greening, 107, 128744. doi: <https://doi.org/10.1016/j.ufug.2025.128744>

Answer:

(In the section of related work, we have revised the article. My ideas are summarized as follows:

1. We briefly explain the technique of extracting entities and relations separately, such as the extraction scheme by Zelenko et al. Then, we introduce joint relation extraction, including the work of Ren et al. and Miwa et al.

2. We add headings and focus on the development of technology for joint extraction of entity relationships based on tagging. First, we introduce the sequence tagging scheme of Zheng [18] and entity splitting scheme of Katiyar[25]. Then, we introduce the ETL model and, based on it, the classic CasRel model. After analyzing the CasRel model, we continue to explain some improvements made by others, such as Zhe [29], Yucheng [27], and Heng [9]. Finally, we introduce our method, which is the tagging-based approach and integrates the ideas of improvements by Zhe, Yucheng, and others.

Additionally, ETL is a relatively classic model, but it is no longer widely used in the development of technology. Among them, BiLSTM is also a classic model, but it is not used in this paper. CasRel is a relatively innovative model in recent years, which has significantly improved performance metrics. Considering the length of the related work section and the advancement of the paper, we decide to remove the equations of ETL and BiLSTM, and only mention the framework of ETL. Many subsequent models have been modified based on CasRel or used as baseline models, so we will focus on introducing the CasRel model and analyzing its advantages and disadvantages.

3. We add heading and introduce the contributions of Chen, Baeksoo Kim, Cho, Dalvi, Zhang, TANG, and others in the plant domain, leading into our goal of improving the performance of joint extraction models.

The corrected part starts from line 157:

Extraction of entities and relations holds significant importance within the realm of natural language processing. In previous approaches, entities and relations are often handled as separate tasks. For instance, Zelenko et al. [28] initially focus on extracting entities from text and then classify the relations between them. While this pipeline model serves its purpose, it is prone to error transfer, whereby mistakes made in entity extraction could affect the accuracy of relation classification.

To address this issue, numerous models for joint extraction of entities and relations have emerged. Ren et al. [24] propose a joint extraction model that relied on a priori features. Building upon this, Miwa and Bansal [13] introduce a parameter-sharing technique that enables entities and relations to be represented by the same encoder, facilitating multi-task learning.

2.1. Tagging-based Joint Extraction

The tagging-based approach is a decoding strategy of joint extraction that designs different tags to reasonably combine entity and relation tasks. Zheng et al. [18] introduce a sequence labeling scheme that includes information on both entities and relations, allowing for the mapping of entities and relations within a unified framework for joint decoding. The scheme directly extracts triples, where each entity is labeled with both a number representing the entity and the type of relations. However, the relations within each dataset are different, and the symbols for these relations need to be redefined in different domains. More importantly, the labeling scheme assumes that each entity has only one relation, making it impossible to identify entities with multiple relations. Katiyar et al. [25] split entities into multiple words and tag each word. However, operating on each token individually does not fully utilize entity boundary information, leading to dispersed information. Subsequently, in 2020, the CasRel model [29] improves extraction performance by incorporating pointer networks. The two classic tagging-based techniques are ETL and CasRel. ETL introduces two distinct strategies to handle entities and relations. In ETL framework, the task is divided into two interconnected subtasks: Head-Entity Extraction (HE) and Tail-Entity and Relation (TER). HE aims to identify the head entities, while TER focuses on identifying tail entities and relations associated with the head entities.

Although ELT employs a logical decomposition strategy to capture the semantic dependencies, it only provides a single recognition result for each entity, leading to omissions when extracting triples for the same entities. Similar to the concept of ELT, CasRel initially extracts head entities, followed by tail entities and relations. However, CasRel integrates pointer networks into the prediction process to tackle the aforementioned challenge. Figure 1 illustrates the architecture of the pointer network model.

Figure 1. Illustration of pointer network.

The pointer network module is used to identify possible entities in the text. It utilizes two distinct binary classifiers to assign a binary label (0/1) to each token. Binary labels serve as indicators for identifying the boundaries of entities. The tag 0 indicates that the current token is not an entity to be recognized, while the tag 1 indicates that the current token is the start or end position of an entity. Equation (1) shows how to determine an entity's start and end positions:

$$p_{i}^{start\_s}=\sigma\left( w_{start}x_{i}+b_{start} \right)$$

$$p_{i}^{end\_s}=\sigma\left( w_{end}x_{i}+b_{end} \right) (1)$$

where variables $w_{sart}$, $b_{start}$, $w_{end}$, and $b_{end}$ are model parameters, $p_{i}^{start\_s}$ and $p_{i}^{end\_s}$ represent the probabilities of the entity's start and end positions, respectively. When recognizing entities, it's common to encounter overlapping entities, where the start positions are the same but the end positions are different. CasRel adopts the proximity matching method, which selects the end label closest to the start position of the overlapping entities as the end position. This method improves the performance of relation extraction, but it also has several limitations. Firstly, the training efficiency is suboptimal. After recognizing entities, Casrel randomly selects one entity for relation prediction, which limits the batch size to be set to one. Secondly, although the pointer network can handle overlapping triples, it generates many zeros, making matrices sparse.

Currently, sequence labeling and pointer network are widely used decoding schemes that can be applied to most scenarios. Thus, a sequence labeling approach is adopted in our research, but types of relations are removed to make it applicable to different kinds of texts. The tagging-based method is essentially multi-task learning. It not only uses different decoding methods but also shares the same encoder parameters in the extraction of entities and relations through parameter sharing. Zhe et al. improve the step of parameter sharing by re-modeling relations as mappings between entity pairs, thereby adding the interaction of relations [29]. Although the model employs parameter sharing method, there is an issue of exposure bias, meaning that the order of entity and relation tasks can lead to inconsistency in the model's parameters during training and inference. Yucheng et al. introduce a concept to address this issue by simultaneously handling the entity tasks and relation tasks [27]. Later, Heng et al. refine the tasks to reduce the complexity of individual tasks, thereby enhancing the performance of parameter sharing [9]. Based on the approaches mentioned above, we divide the task into different subtasks, and simultaneously extract relations and entities. Next, we make two adjustments. Adjustment to the word embeddings enhances text representation, enabling the model to balance different tasks and stabilize parameters. Adjustment to relation task improves the accuracy of relation extraction, which can better interact with other tasks and reduce the accumulation of errors from one stage to subsequent tasks.

2.2 Relation Extraction in Plant Texts

Relation extraction based on deep learning has achieved great result. In plant texts, relation extraction is also a significant research direction, with extensive application prospects in assisting botanical research and other fields. Chen et al. [38] examine how specific landscape practices, leave a lasting imprint on urban flora, identifying 211 spontaneous plant species in 169 genera from 82 families. Baeksoo Kim et al. [2] develop a corpus focusing on plants and their treatment of diseases, expanding the application range of plant data. [2]. Cho et al. [10] extract plant entities based on word embeddings and Dalvi et al. [39] develop a robust deep CNN model to identify plant entities, enhancing the accuracy of the model in identifying medicinal plants related to skin health, specifically their leaves, trunks, and seeds. But they do not take into account the impact of relations. Zhang et al. [30] employ an approach combining BiLSTM and CRF to extract entities and relationships, but the accuracy is suboptimal. Tang et al. [40] propose an entity recognition model and a relation extraction model, which are embedded with ALBERT, solving the problem of having poor ability of context fusion and computational efficiency. However, this method falls under the category of pipeline extraction models. Pipeline models are easier to implement but overlook the dependencies between entities and relations. Later, Zhu et al. [41] propose a scalable natural language annotation framework. The framework is a joint extraction model that employs convolutional neural networks to extract features at different depths and uses inter layer feature fusion to enhance feature representation capabilities. Compared to pipeline models, joint extraction models simultaneously extract entities and relations, naturally leveraging their interdependencies. In this paper, we will continue to explore the potential of joint extraction models in plant texts scenarios, improving the performance of joint extraction models. ）

5) A table of symbols used in the paper be presented with description for better understanding.

Answer:

(In the section of Introduction. We provide a symbol table in our paper, along with corresponding descriptions, to facilitate better understanding.

| Serial number | Symbol | Description |
| --- | --- | --- |
| 1 | $p_{i}^{start\_s}、p_{i}^{end\_s}$ | The prediction results of the start and end positions of entities in a pointer network. |
| 2 | $x_{i}$ | Input vector of a pointer network. |
| 3 | $T(S)$ | Relational triple extracted from a sentence. |
| 4 | $h,t$ | Head entity and tail entity in a relational triple. |
| 5 | $r$ | Relation between the head entity and the tail entity. |
| 6 | $n$、$n_{r}$、$n_{r}^{pot}$ | The total number of entities, the number of potential relations and the number of actual relations. |
| 7 | $p_{rel}$ | The probability of predicted relations. |
| 8 | $x、\Delta x$ | The input vector of the model and perturbation. |
| 9 | $g$ | The derivative of loss at the word embeddings. |
| 10 | $e$、$e^{avg}$ | The input of predicting relations and the result of the operation of average pooling. |
| 11 | $p_{i,j}^{sub}、p_{i,j}^{obj}$ | Prediction of {B,I,O} for the current entity in sequence labeling. |
| 12 | $p_{i_{sub ,} j_{obj}}$ | The value of the element in global matrix. |

)

6) Conclusions are presented however future research work should also be proposed in this section and the title of the section can be changed to Conclusion and Future work

Answer:

(We have modified the title here and added the content of the future work. The title is: Conclusions and Future Work.

The corrected part starts from line 793:

In this paper, we explore joint extraction model based on plant dataset. Analyzing the text, 13 attribute categories are defined and plant entities are fixed as the head entities to narrow the scope of extraction. To alleviate the issues of error amplification and unstable parameter updates in multi-task parameter sharing, this paper proposes a model called Bwdgv. The model introduces perturbations in the word embedding layer and employs a multi-level information fusion strategy for relation prediction. We use accuracy, F1 score, and recall as metrics to evaluate model's performance. By comparing with other models such as CasRel, our model demonstrates superior extraction performance and excels in handling complex scenarios, such as overlapping patterns and sentences with different numbers of triples. In addition, based on the insights gained from this research, future attempts may involve improving entity relation ex-traction performance by incorporating additional semantic features in the relation ex-traction phase, optimizing perturbation for specific subtasks, or exploring alternative feature fusion methods in entity relation pair matching. Finally, these triples extracted from plant texts can be applied in fields such as knowledge graph to improve the rapid retrieval of plant information.）

Reviewer #4:

1、The three-stage joint extraction framework proposed in this article has achieved significant results in SEO overlap mode, but the discussion of existing models (such as ETL Span, OneRel, etc.) in the introduction and related work sections is relatively brief. Suggest adding comparisons with these latest models and clearly pointing out the technical and performance differences between our method and these models. This will help readers understand the innovation and advantages of the proposed method.

Answer:

(Considering that we have already analyzed the principles and defects of Casrel and ETL in the related work section, we decide to compare several models in the introduction.

The corrected part starts from line 112:

Based on this, we review several models. The ETL model [36] and the CasRel model [29] both first extract the head entity, and then simultaneously extract the rela-tion and the corresponding tail entity based on the head entity. However, the relations between the two entities in the ETL model is unique, which would result in missing triples with different relations. CasRel solves this issue by extracting the tail entity separately for each relation, but the number of relations is large. In comparison, our model optimizes the extracting of relations, namely the classification of plant attributes, thereby reducing the redundancy of relations. For the TPLink model [27], it uses many matrices and extracts the head and tail entities with different matrices, which dissociates the dependency relations between entities. On this basis, OneRel [35] pro-poses a method that uses a matrix for each relation and extracts triples from this matrix. However, compared to our model, OneRel has more relations, which can generate more negative samples.)

2、The integration logic of BERT wwm and adversarial training in Section 3.1 needs further clarification, especially on why perturbations are added at the word embedding layer instead of being processed at other layers, and how this design mitigates the negative impact of parameter sharing. In addition, it is recommended to further analyze the specific impact of this design on the stability of model training.

Answer:

(We expand the explanation of the integration logic of BERT wwm with adversarial training. First, we summarize the actions taken to mitigate the negative impact of parameter sharing, and then introduce BERT-wwm and the addition of perturbations in sequence. When adding perturbations, we explain why the perturbations are added at the word embedding layer rather than processed in other layers. Finally, in the gradient update section, we analyze the impact of gradient calculation on model training stability.

The corrected part starts from line 301:

Although parameter sharing helps to reduce the number of parameters in the model, it may also lead to a decrease in the model’s ability to distinguish between errors. To mitigate the negative impact of parameter sharing, the following operations are employed. Firstly, BERT-wwm is used to more accurately represent text information, reducing the occurrence of errors. Secondly, perturbations are added to pro-mote the model to learn more general features. Due to parameter sharing, perturbations can be more effectively propagated to other layers, forcing the model to learn subtle changes in the data. Consequently, the model can enhance its adaptability to changes in input, reducing the propagation of errors.

……………………

The text representations are used as input for subsequent subtasks. Considering the accuracy of the information and the stability of the training process, perturbations are introduced into the model. Perturbations can be implemented through data augmentation, but text data does not have operations like image flipping or cropping. The vectors converted from text are essentially one-hot vectors. A one-hot vector has a value of 1 at only one position and 0 everywhere else. The distance between the any two vectors is always √2. Therefore, adding perturbations to text vectors also has no effect. Manually adding characters is a way to introduce perturbations, but this method is inefficient and prone to semantic ambiguity. According to the improvements made by Giannis et al. on the LSTM model [31], fast gradient method (an adversarial training method) is adopted to construct perturbations. In the field of deep learning, the term "adversarial" typically refers to the generation of adversarial networks and adversarial samples [37]. Adding perturbations is equivalent to constructing adversarial samples and the procedure of identifying adversarial samples is commonly referred to as adversarial training. Adversarial training serves as an effective defense mechanism in the realm of adversarial attacks. This defensive approach is guided by Equation (3):

………………..

The core of adversarial training is to find an appropriate ∆x. Word embedding layer, as the direct interface between the model and input text, serve to map words into dense vectors. Adding perturbations here can directly affect the model’s understanding of vocabulary. In addition, compared to adding perturbations in deeper layers, perturbations in the word embedding layer are easier to control and do not have impact on the other structures of the model. Thus, we add perturbations in the word embedding layer. As shown in Fig. 3.

The perturbation ∆x is directly added to the word embedding matrix. The input is first converted into a one-hot vector, and then transformed into a text vector through the word embedding layer. At this point, different samples will share the same perturbations, making the implementation easier. Finding the location where the perturbations need to be added, and then the perturbations can be calculated. The goal of perturbations ∆x is to maximize L(x+∆x,y;θ), and the direction of gradient ascent for L is exactly the direction in which L increases. We view the gradient of L to be proportional to ∆x. The calculation of the perturbations is shown in Equation (4):

where g is the derivative of L(x+∆x, y;θ) at the word embeddings, ε is the proportional coefficient, normalization is used to prevent perturbations from becoming too large. When constructing perturbations, the first step is to compute the loss of forward propagation and record the gradients of backward propagation. At this point, the gradients taken from the word embeddings, are used to calculate the perturbations according to equation (6). And then, the perturbations are added to the word embedding to construct a new input x+∆x. Finally, the loss of forward propagation and the gradient of backward propagation are calculated once again. It should be noted that in the first training, the gradient does not need to be updated. In the second training, the gradient to be updated is the sum of the gradients calculated in the first and second computations. At this time, the model learns the features of both normal samples and adversarial samples simultaneously, better adapting to data changes and reducing fluctuation in the training. When updating the gradient, the input of word embeddings should revert to the original x. As the update without considering disturbances, the model reduces its reliance on adversarial samples, ensuring the stability of parameters. Based on this, the model learns how to resist noise at the input level and ensures the stability of the training process.

)

3、In the experimental section, it is necessary to supplement the comparison of the models in Table 2 and clarify whether the pre trained models used are consistent, as well as whether the hyperparameter settings are the same, to ensure the comparability of the experimental results. Meanwhile, the clarity of the experimental results and model architecture diagrams is poor. It is recommended to increase the resolution and annotation clarity of the charts to ensure their ease of interpretation.

Answer:

(

1. Add a model comparison description in section 4.1 Experimental Data and Setting.

2. We merge table 2 and table 3 into one table for description, and rename it to table 3. This table only describes the comparison results of the experiment.

3. we upload the images to the journal’s designated website https://pacev2.apexcovantage.com/ to verify and modify, so as to meet the image requirements.

The corrected part starts from line 576:

1.

The model is evaluated from two perspectives: the number of triples and the over-lapping patterns of triples. The evaluation metrics are accuracy, precision, and recall. This experiment is conducted using the PyTorch framework, encompassing the pre-training model BERT and other neural networks. The hardware configuration for this experiment consists of an AMD EPYC 7371 CPU with 28GB of operating memory, ac-companied by an RTX A5000 GPU with 24GB of memory. As for the software configuration, the experiment utilizes the PyTorch 1.7.0 framework, Python 3.8 programming language, and is operated within the Ubuntu 18.04 environment. All key parameters within the experiment are set as follows: the maximum text length is restricted to 512, the adversarial training perturbation value is set at 0.005, the learning rate of the pre-trained model is 0.0001, and early stopping is implemented after 30 epochs. In the experiment, all compared models are based on the pre-trained model, BERT-base. Their hardware configuration, text length, and early stopping are also kept the same. Due to structural limitations, CasRel can only be set to a batch size of 1. The other models maintain the same batch size as our model.

The corrected part starts from line 633:

2.

Our model outperforms other models in terms of precision, recall, and F1-score. Taking plant dataset as an example, the F1 score of our model obtains 6.9% improvements than CasRel, 3.2% improvements than OneRel, 2.9% improvements than TPLinker, 2% improvements than GRTE, 0.4% improvements than UniRel, and 1.4% improvements than PRGC. In training, entities in CasRel model are real labels, but dur-ing inference, the prediction of tail entities depends on the identification of head entities. Errors in the head entities can lead to a decline in extraction accuracy. In comparison, the multi-task strategy of Bwdgv can enhance the interaction between subtasks, resulting in better extraction accuracy. TPLink model constructs three matrices to ex-tract triples. But these matrices contain redundant information and have limited ability to generalize when extracting entities of varying lengths. GRTE improves global features based on TPLinker, but has low decoding and inference efficiency. Compared with these two, Bwdgv only constructs a matrix in entity pair matching, and optimizes entity recognition, enhancing its generalization ability. PRGC tends to recognize relations by remembering their positions rather than their semantics. In contrast to PRGC, Bwdgv enhances the semantic information of relations through multi-level infor-mation fusion. UniRel focuses on the integration of relational semantics, achieving mu-tual inference between entities and relations. In comparison, Bwdgv not only focuses on relations but also considers the balance between tasks. OneRel model employs a one-step decoding method to directly extract triples, but its design is more complex. Experimental results obtained on a single dataset may be accidental. To verify the model's performance in different data scenarios, we conduct experiments on Chinese Medicine Information Extraction dataset (CMeIE). The dataset contains two corpora: one is a pediatric training corpus, and the other is a common disease training corpus. It includes 14,339 training sentences, 4,482 test sentences, and 53 predefined relation types. Despite the increased complexity of the dataset causing a decline in the performance of all models, our model still surpasses other models in terms of precision and F1-score. This demonstrates the reliability of our model in different textual scenarios. Overall, the excellent performance of Bwdgv is mainly attributed to its optimized entity recognition strategy, enhanced semantic information processing capabilities, and efficient multi-task strategy. Meanwhile, the performance of Bwdgv in plant dataset during training is shown in Figures 8-10.）

4、Finally, it is recommended to quantify the specific impact of adversarial training on F1 score in the ablation experiment, in order to more clearly demonstrate the contribution of adversarial training to model performance and help readers understand its effectiveness.

Answer:

(We adjust the perturbation value to quantify the F1 score of adversarial training under different perturbation values. We set Δx to different values to validate the effectiveness of the model.

The corrected part starts from line 677:

|  | Models | *prec* | *Rec* | *F1* |
| --- | --- | --- | --- | --- |
| plant dataset | Bwdgv(adjustment to word embedding layer, $\Delta x=0.005$) | 88.1 | 89.3 | 88.7 |
|  | Bwdgv(adjustment to word embedding layer, $\Delta x=0.05$) | 88.3 | 89.5 | 88.9 |
|  | Bwdgv(adjustment to word embedding layer, $\Delta x=0.5$) | 88.3 | 89.8 | 89.1 |
|  | Bwdgv(optimization in relation prediction) | 87.8 | 89.2 | 88.5 |
|  | Bwdgv(all,$\Delta x=0.005$) | 88.5 | 89.8 | 89.2 |

First, we only keep the adjustment to word embedding component and observe the changes in model's performance. The accuracy, precision, and F1-score of the model still perform well. This demonstrates the effectiveness of the adjustment to the word embedding component. Perturbation introduced in the word embedding layer is con-structed by adversarial training to reduce overfitting of tasks. When facing complex contextual information, this component enhances the model's generalization performance by encouraging the model's gradients to continually move beyond a certain range, thereby avoiding getting stuck in local optima. To measure the performance of the model under different perturbations, we adjust the value of Δx. At this point, the F1 scores of the models have all improved, indicating that introducing appropriate perturbations helps to enhance the model’s performance. When Δx is 0.5, the model per-forms better in terms of F1-score. When Δx decreases to 0.005, Recall decreases from 89.8% to 89.3%, and Precision decreases from 88.3% to 88.1%, indicating that the perturbations have a more noticeable impact on the model’s recall. Therefore, slightly larger perturbations can help the model identify more positive samples. Following, we only keep the optimization component in Relation Prediction and the performance of the model still remains great. This indicates that it is important for the overall success of the model. This component enhances the accuracy of relation task by integrating multi-layer information, thereby effectively reducing the propagation of errors in downstream tasks. If the model relies solely on basic module of relation embeddings, which typically only captures shallow co-occurrence relations, it will be difficult to effectively identify and utilize context information at different levels, leading to a decline in performance in subsequent tasks. By introducing these two components, the performance of the model in multi-task learning has been enhanced.

)
